# 9-Aminoacridine Inhibits Ribosome Biogenesis by Targeting Both Transcription and Processing of Ribosomal RNA

**DOI:** 10.3390/ijms23031260

**Published:** 2022-01-23

**Authors:** Leonid Anikin, Dimitri G. Pestov

**Affiliations:** 1 Department of Cell Biology and Neuroscience, Rowan University School of Osteopathic Medicine, Stratford, NJ 08084, USA; Leonid.Anikin@wuxiapptec.com; 2 Graduate School of Biomedical Sciences, Rowan University School of Osteopathic Medicine, Stratford, NJ 08084, USA

**Keywords:** ribosome biogenesis, rRNA transcription, RNA processing, nucleolus

## Abstract

Aminoacridines, used for decades as antiseptic and antiparasitic agents, are prospective candidates for therapeutic repurposing and new drug development. Although the mechanisms behind their biological effects are not fully elucidated, they are most often attributed to the acridines’ ability to intercalate into DNA. Here, we characterized the effects of 9-aminoacridine (9AA) on pre-rRNA metabolism in cultured mammalian cells. Our results demonstrate that 9AA inhibits both transcription of the ribosomal RNA precursors (pre-rRNA) and processing of the already synthesized pre-rRNAs, thereby rapidly abolishing ribosome biogenesis. Using a fluorescent intercalator displacement assay, we further show that 9AA can bind to RNA in vitro, which likely contributes to its ability to inhibit post-transcriptional steps in pre-rRNA maturation. These findings extend the arsenal of small-molecule compounds that can be used to block ribosome biogenesis in mammalian cells and have implications for the pharmacological development of new ribosome biogenesis inhibitors.

## 1. Introduction

Ribosome biogenesis, a resource- and energy-demanding process of generating new ribosomes, is indispensable for cell growth and proliferation. In mammalian cells, the synthesis of cytoplasmic ribosomes starts in the nucleolus with RNA polymerase I (Pol I)-mediated transcription of long polycistronic transcripts, termed 47S pre-rRNA, which undergo multistep processing to 18S, 5.8S, and 28S rRNAs, concomitant with the assembly of the ribosome structure [1]. Owing to the complexity of the ribosome assembly mechanisms, the synthesis of new ribosomes is sensitive to many types of metabolic perturbations and stress [2]. Cellular responses to defects in ribosome biogenesis have been implicated in human pathologies including bone marrow failure, developmental abnormalities, and cancer [3,4,5]. 

In recent years, there has been growing interest in targeting ribosome biogenesis as an anticancer therapeutic strategy [6,7,8]. One rationale for this is that in order to proliferate rapidly, tumor cells must upregulate the synthesis of ribosomes, making these cells susceptible to ribosome biogenesis inhibition [9,10]. In addition, inhibitors of ribosome biogenesis activate the nucleolar stress response [11,12], which may potentiate cytotoxic effects by promoting cell death in certain cancer cells [8]. Conversely, cytostatic responses to nucleolar stress in nonmalignant cells could be beneficial by rendering these cells resistant to S phase-specific anticancer drugs [13]. 

To date, targeting ribosome biogenesis in mammalian cells with small molecules has been achieved primarily through drugs that inhibit Pol I transcription of pre-rRNA [14]. Actinomycin D (ActD) is a DNA-binding antibiotic that has been widely used as an inhibitor of Pol I transcription based on early observations showing the hypersensitivity of cellular pre-rRNA synthesis to low ActD concentrations [15]. However, later work showed that at the concentrations typically used in cell culture experiments, ActD does not prevent Pol I initiation, promoting instead an accumulation of prematurely terminated transcripts [16,17], which engage the nucleolar RNA degradation machinery [18,19]. 

Among several newer inhibitors of Pol I transcription developed in recent years, CX-5461 [20] has been shown to arrest Pol I in transcription initiation complexes [21]. This mode of action is accompanied by replication stress, DNA damage [22,23], and activation of the cGAS–STING signaling pathway [24]. BMH-21 was reported to repress Pol I transcription through preferential binding to GC-rich sequences present in ribosomal DNA genes, degradation of a Pol I catalytic subunit [25], and inhibition of transcription elongation [26]. Subsequent in vitro analysis has shown that BMH-21 inhibits initiation, clearing of the promoter, and elongation by the yeast Pol I enzyme [27].

Recent evidence suggests that transcription by Pol I is broadly sensitive to DNA intercalators and groove binders [28]. Acridines represent a class of three-ring heterocyclic compounds that are best known for the ability to reversibly bind to DNA via intercalation [29]. This property is especially notable in aminoacridines, many of which are present in a cationic form at physiological pH [30]. Several acridines have also been demonstrated to inhibit RNA polymerases in vitro [31,32,33]. More recently, high-throughput screens of chemical libraries identified derivatives of acridines and structurally related benzonaphthyridines (such as CID-765471/BMH-22) among nucleolar disruptors and Pol I transcription inhibitors [34,35]. The aminoacridine derivative quinacrine, used clinically as an antimalaria drug, was recently reported to induce nucleolar stress in cultured cancer cells [36]. These effects on ribosome biogenesis suggest the potential of acridines for therapeutic repurposing. 

In this study, we investigated the effects of a prototypic aminoacridine compound, 9-aminoacridine (9AA), on ribosome biogenesis in mammalian cells. We report here that both transcription and maturation of pre-rRNA are inhibited rapidly after cell exposure to 9AA. We also show that 9AA readily binds to RNA, which likely causes interference with post-transcriptional pre-rRNA processing. These results identify 9AA as an antagonist of ribosome biogenesis with a unique set of properties. In addition, these findings suggest that aminoacridine-based compounds are attractive candidates for the discovery of new ribosome biosynthesis inhibitors.

## 2. Results

### 2.1. 9AA Is a Potent Inhibitor of Pre-rRNA Transcription

**Figure 1:**
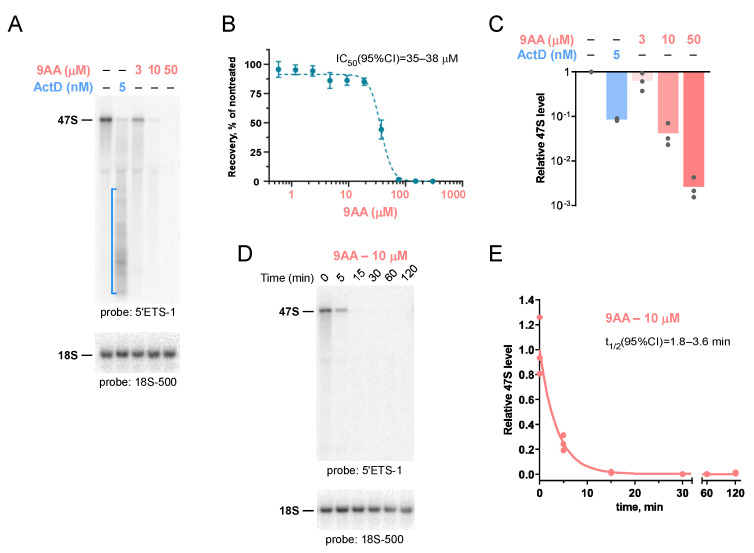
9AA reduces 47S pre-rRNA levels in NIH 3T3 cells. (**A**) Northern hybridization of RNA from cells treated with different concentrations of 9AA or ActD for 2 h. Products of Pol I transcription were visualized with probe 5’ETS-1 (top panel); the same blot was then rehybridized with probe 18S-500 (bottom panel) to control loading. The bracket marks the smear of incomplete Pol I transcripts accumulating after ActD treatment. (**B**) The number of viable, metabolically active cells determined using an Alamar blue assay at 72 h after a 2 h 9AA treatment, relative to untreated cells. *n* = 5; error bars indicate s.d. IC_50_ was estimated using a variable-slope dose-response model. (**C**) Relative abundance of the primary 47S pre-rRNA transcript. Hybridization signal of full-length 47S was normalized by 18S in the same lane to correct for loading variation; bars show mean values obtained from 3 replicate cell cultures. (**D**) Representative time course of a 10 μM 9AA treatment. Northern hybridizations were performed as in (**A**). (**E**) Change in the primary 47S transcript levels over time after cell exposure to 9AA. Data from 3 replicate cell cultures were fit to a one-phase exponential decay equation.

To investigate whether 9AA affects pre-rRNA transcription, we examined pre-rRNA transcript levels in mouse 3T3 cells after treating the cells for 2 h with 9AA and using ActD in parallel as a benchmark Pol I inhibitor (Figure 1A). We tested 9AA concentrations that were well-tolerated (3–10 µM, >80% cell recovery after a 2 h exposure to 9AA) and a cytotoxic 50 µM dose (<20% cell recovery, Figure 1B). The oligonucleotide probe 5’ETS-1 used in the transcript analysis (Figure 1A) hybridizes with the 5’ region of nascent Pol I transcripts, quickly removed during their normal post-transcriptional processing and thus absent in all but the earliest rRNA precursors ([37], Figure S1). Consistent with previous studies [17,18,19], treatment of cells with 5 nM ActD inhibited elongation by Pol I, leading to a decreased synthesis of the full-length 47S pre-rRNA and accumulation of incomplete transcripts (Figure 1A). An inhibitory effect of 9AA on Pol I transcription was evident at all tested (3–50 µM) concentrations of 9AA in the cell medium; however, no accumulation of short transcripts was detected (Figure 1A). By quantifying levels of the full-length 47S transcripts (Figure 1C), we estimate that 10 μM 9AA led to a ~24-fold reduction in the 47S pre-rRNA following a 2 h incubation (mean value ± s.e.m.: 4.2 ± 1.4% relative to the 47S level in untreated cells), which was comparable with the reduction achieved with 5 nM ActD (8.6 ± 0.4%). 

To determine how rapidly a 9AA treatment can shut down pre-rRNA transcription, we next examined 47S pre-rRNA levels at different time points after cell exposure to 10 μM 9AA. As shown in Figure 1D,E, a > 90% drop in the 47S pre-rRNA level occurred within 10 min after 9AA was added to the medium. Thus, 9AA is highly efficient in reaching the nucleolus from the extracellular environment and can block RNA Pol I transcription within minutes after its initial contact with cells. An efficient inhibition of Pol I transcription by micromolar concentrations of 9AA was observed in other tested cell lines. As an example, 10 μM 9AA induced a > 100-fold reduction in the 47S pre-rRNA levels in the human fibrosarcoma HT1080 cells using the same 2 h treatment, although these cells exhibited a somewhat slower 47S decay kinetics than mouse 3T3 cells (Figure S2A–D).

### 2.2. Dose-Dependent Effects of 9AA on Nucleolar Integrity

**Figure 2:**
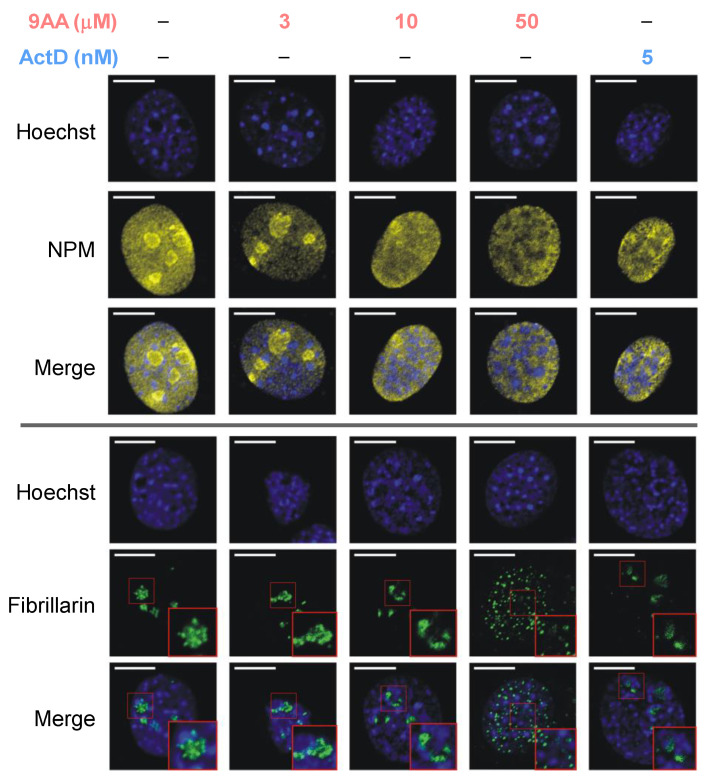
Treatment of 3T3 cells with 9AA induces nucleolar disruption. Cells were treated with the indicated concentrations of 9AA or ActD for 2 h. Nuclei were stained with Hoechst 33342; NPM and fibrillarin were detected by immunofluorescence staining. Representative confocal microscopy images are shown; insets show higher magnification of the indicated areas of the nucleus. Scale bar, 10 μm.

Transcription of pre-rRNA and most steps in ribosome assembly occur inside the nucleolus. The establishment and maintenance of the nucleolar structure are intrinsically associated with ongoing Pol I transcription [38]. To examine the consequences of 9AA treatment on nucleolar integrity, we probed the localization of nucleophosmin (NPM) and fibrillarin, two well-characterized markers of the granular and dense fibrillar compartments of the nucleolus, respectively. In untreated interphase 3T3 cells, immunofluorescence microscopy analysis showed a diffused nucleolar accumulation of NPM, while fibrillarin displayed a structured intranucleolar distribution (Figure 2), consistent with the known localization patterns of these proteins [39]. As further illustrated in Figure 2, no gross changes in the distribution of either NPM or fibrillarin were apparent in the nucleoli of cells treated with 3 µM 9AA, a dose that is moderately inhibitory for transcription of pre-rRNA in these cells. In contrast, treatment with 10 μM 9AA, which strongly inhibits 47S pre-rRNA transcription, caused marked changes in the localization of both proteins: NPM largely relocated to the nucleoplasm, while fibrillarin displayed characteristic segregation into nucleolar caps [39]. Similar changes in the nucleolus were observed with 5 nM ActD. Interestingly, further increasing the dose of 9AA to 50 µM led to a dramatic nuclear redistribution of fibrillarin in many treated cells, producing a pattern of small bodies scattered across the nucleus (Figure 2). These data show that 9AA exerts dose-dependent effects on the nucleolus, with the typical remodeling of nucleolar architecture observed at 10 μM when transcription of 47S pre-rRNA is repressed. The loss of nucleolar fibrillarin observed at a higher dose of 9AA implies a further destabilization of the molecular interactions critical for maintaining the nucleolar structure, beyond the Pol I transcriptional block.

### 2.3. 9AA Can Inhibit Post-Transcriptional Steps in the Synthesis of Nascent Ribosomes 

**Figure 3:**
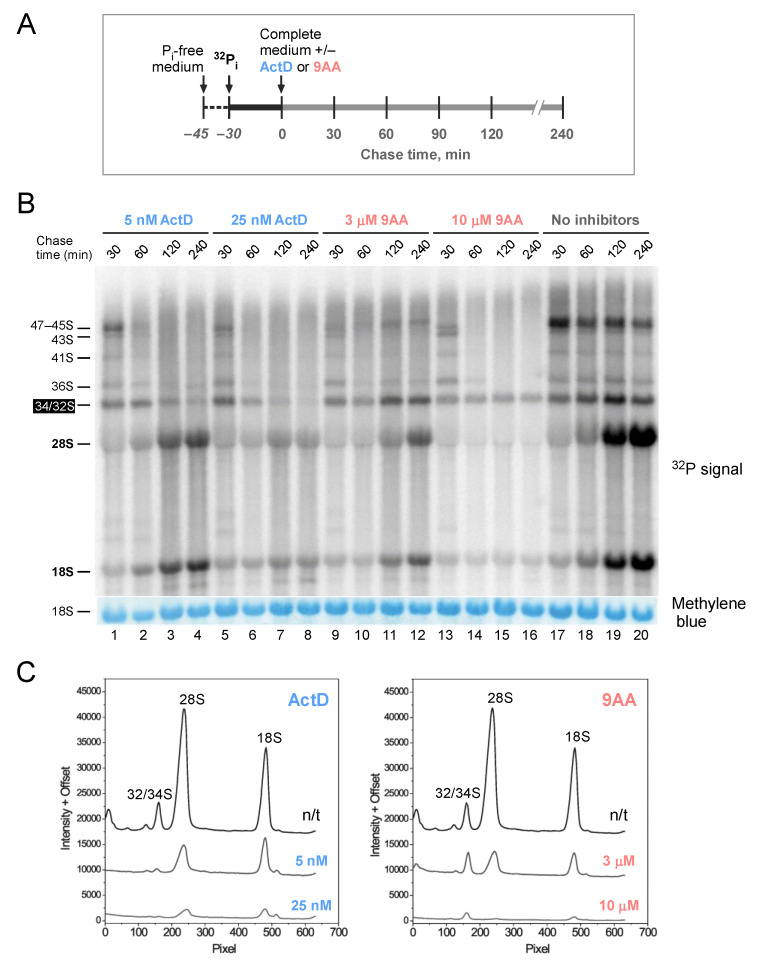
Metabolic labeling analysis of pre-rRNA maturation. (**A**) Cells were incubated in phosphate-free medium for 15 min, [^32^P]-orthophosphate was added for 30 min, after which the labeling mix was removed, and the cells were incubated in complete growth medium with or without inhibitors for different times. (**B**) The isolated total cellular RNA was resolved on an agarose gel, transferred to a nylon membrane, and analyzed by phosphor imaging. At the bottom, 18S stained on the same membrane is shown to indicate loading. See Figure S1 for a diagram of mouse rRNA precursors. The highlighted 34S and 32S pre-rRNAs are further discussed in the text. (**C**) Radioactive traces of gel-separated RNA from cells that were treated for 240 min with the indicated concentrations of ActD and 9AA. Traces were obtained by phosphor imaging; pixel numbers indicate distance from the gel origin (each pixel unit is 0.1 µm).

To better understand 9AA effects on the nucleolar biosynthetic processes, we next examined post-transcriptional maturation of pre-rRNA by incubating cells with [^32^P]-orthophosphate for 30 min followed by incubation with or without inhibitors for various times up to 240 min (Figure 3A). In the absence of added inhibitors, the baseline pattern of [^32^P] labeling (Figure 3B, lanes 17–20) shows increasing label incorporation into 28S and 18S rRNAs during the 240 min chase, indicative of productive processing of pre-rRNA into mature rRNAs. The continuing transcription of pre-rRNA during the chase also results in the labeling of rRNA precursors due to the [^32^P] present in the cellular phosphate pool. When cells were treated with 5 nM ActD (Figure 3B, lanes 1–4), new pre-rRNA transcription was blocked and the already synthesized precursors were gradually converted into mature 28S and 18S rRNAs. This was also the case when cells were treated with 25 nM ActD, although the overall efficiency of rRNA maturation declined at this higher ActD concentration (Figure 3B, lanes 5–8), as could also be seen from the quantitative measurements of the radioactivity incorporated into 28S and 18S rRNAs (Figure 3C, left panel).

The rRNA labeling pattern after treatment of cells with 9AA was distinct from that in ActD-treated cells. Although 3 μM 9AA only modestly reduced pre-rRNA transcription (Figure 1C), [^32^P] incorporation into mature rRNAs was unexpectedly lower than in the presence of 5 nM ActD (Figure 3B, lanes 9–12, Figure 3C). Increasing the 9AA concentration to 10 μM completely abolished the label incorporation into 28S or 18S rRNA (Figure 3B, lanes 13–16). A prominent band corresponding to comigrating 34S and 32S pre-rRNAs was persistently present throughout the 240-min chase after 9AA treatments, suggesting compromised pre-rRNA maturation. Further increasing the 9AA dose to 50 μM led to an additional accumulation of pre-rRNAs in the 47S-43S range (Figure S3A), indicating inhibition of early pre-rRNA processing. Together, these data indicate that in addition to being an inhibitor of pre-rRNA transcription, 9AA interferes with post-transcriptional steps of ribosome synthesis.

### 2.4. Effects of 9AA on Processing of Pre-rRNA

**Figure 4:**
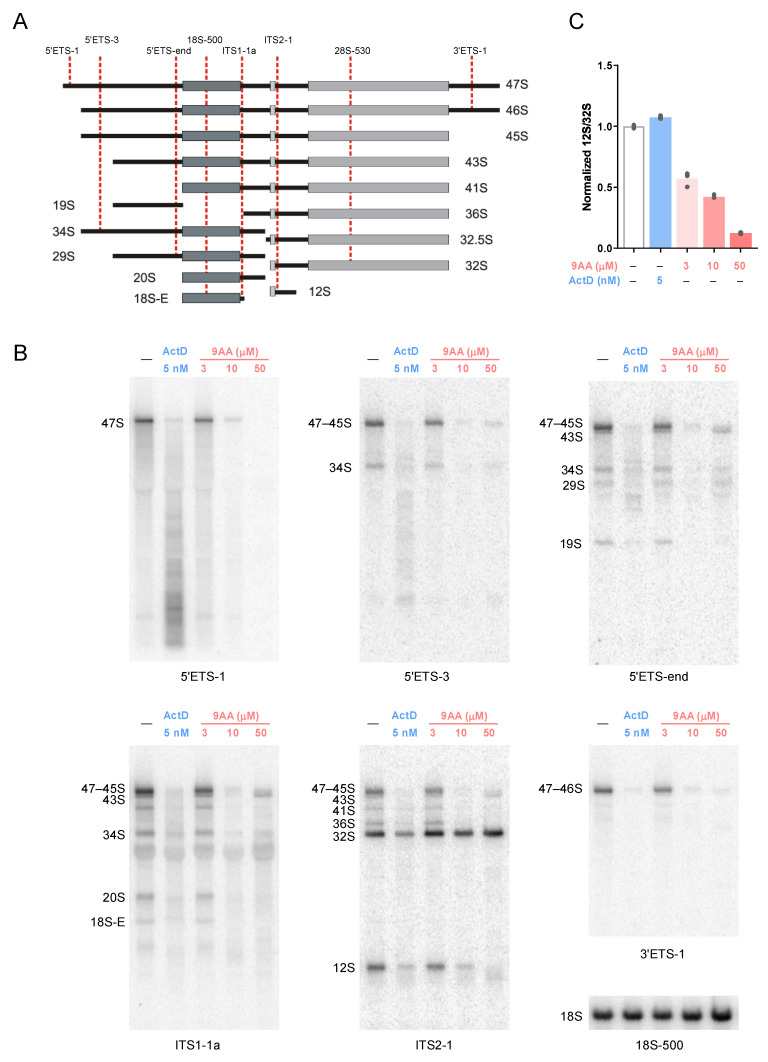
9AA inhibits pre-rRNA processing. (**A**) Major rRNA precursors in mouse cells. Oligonucleotide probes used for the detection of pre-RNAs via hybridizations are diagrammed at the top. Probe sequences are listed in Table S1. (**B**) Hybridization analysis of pre-rRNA. NIH 3T3 cells were treated with ActD or 9AA for 2 h. Total cellular RNA was extracted, resolved on a 1% agarose gel, and transferred onto a nylon membrane. The membrane was sequentially hybridized with the indicated probes. The experiment was repeated three times with similar results; representative images are shown. (**C**) Effects of 9AA and ActD on the efficiency of 32S conversion to 12S pre-rRNA. The ratios of the 12S and 32S pre-rRNA hybridization signals in the same lane were determined by phosphor imaging in three replicate cell cultures; bars depict mean values.

To examine the effects of 9AA on pre-rRNA processing in more detail, we performed northern hybridizations using probes that detect a range of rRNA precursors (Figure 4A). As expected, treatments with 5 nM Act D and 10 μM 9AA, which strongly inhibited transcription of 47S pre-rRNA (Figure 1A,C), caused diminished levels of most processing intermediates (Figure 4B). Paradoxically, further increasing 9AA concentration to 50 μM led to elevated levels of several pre-rRNA species including 43S, 34S, and 29S (Figure 4B), indicating that the higher 9AA dose inhibited processing of the pre-rRNAs that existed in the cells at the time when they were exposed to the drug. This was in agreement with the labeling pattern of 47-43S in the presence of 50 μM 9AA (Figure S3A). 

Northern analysis also showed that 32S pre-rRNA was inefficiently processed. During normal pre-60S ribosome maturation, the 32S pre-rRNA is cleaved to yield 12S and 28.5S pre-rRNAs [1]. To evaluate the efficiency of this cleavage, we quantified the relative abundance of the 12S and 32S pre-rRNAs in each lane of the blot using probe ITS2-1 that hybridizes with both pre-rRNAs (Figure 4A,B). As quantification of the data has shown (Figure 4C), 9AA treatment causes a dose-dependent decline in the 12S/32S pre-rRNA ratio, indicating an inhibition of the 32S to 12S processing. Notably, even the lowest tested 9AA concentration, 3 μM, already caused a ~2-fold drop in the 12S/32S ratio compared to untreated cells, attesting to the high sensitivity of this processing step to 9AA. By contrast, there was no significant change in the 12S/32S ratio in ActD-treated cells, indicating that the effects observed for 9AA were not a general consequence of reduced pre-rRNA transcription. A time-course analysis of 32S and 12S levels corroborated these results by showing persistent levels of 32S pre-rRNA after a 9AA treatment (Figure S3B). The inhibitory effect of 9AA on 32S pre-rRNA processing was reproduced in human HT1080 cells (Figure S2E,F). 

Collectively, these data establish that in addition to inhibitory effects on pre-rRNA transcription, 9AA inhibits pre-rRNA processing in a dose-dependent manner. Processing of 32S pre-rRNA, required for the synthesis of new 60S ribosomal subunits, is especially sensitive to 9AA. However, at a very high (50 μM) dose of 9AA, pre-rRNA processing appears broadly impaired, leading to accumulation of multiple pre-rRNAs. Notably, the nucleolar structure becomes severely disrupted at this 9AA concentration, as judged by the loss of fibrillarin from the nucleolus (Figure 2).

### 2.5. Binding of 9AA to RNA

**Figure 5:**
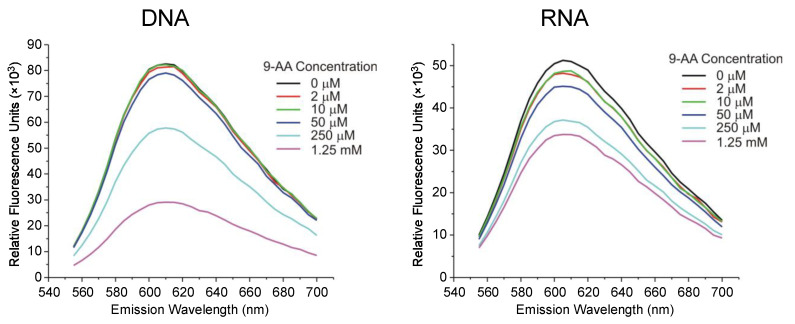
Fluorescent intercalator displacement. Emission spectra of EtBr bound to salmon sperm DNA and baker’s yeast RNA in the presence of increasing concentrations of 9AA; λex = 525 nm.

Although acridines are primarily considered to be DNA intercalators, several acridines, such as acridine orange [40] and proflavin [41], have been noted for binding to RNA. We asked whether 9AA was capable of binding to RNA, which could explain its prominent effects on pre-rRNA processing. We used a fluorescent intercalator displacement assay [42] in which the binding of small molecules to nucleic acids can be detected by changes in the fluorescence of ethidium bromide (EtBr). The intensity of EtBr fluorescent emission is enhanced by binding to DNA and RNA [43,44]; therefore, dissociation of EtBr from the nucleic acids by competing compounds will decrease the observed fluorescence intensity. Although 9AA is a fluorescent molecule itself, the excitation and emission peaks for 9AA (380, 450 nm) and EtBr (525, 605 nm) are sufficiently far apart to allow measurements of EtBr fluorescence in the presence of 9AA. The obtained fluorescence emission spectra (Figure 5) demonstrated a dose-dependent decrease in EtBr binding to RNA and DNA in the presence of 9AA, consistent with 9AA binding to both nucleic acids in a manner that causes EtBr to dissociate. Thus, 9AA binds to RNA effectively, supporting the notion that the observed effects of 9AA ribosome biogenesis could be mediated by the drug’s interaction with RNA molecules.

## 3. Discussion

In this study, we demonstrate the potent ability of 9AA to inhibit both transcription and post-transcriptional processing of pre-rRNA in cultured mouse and human cells. These properties make 9AA a useful tool for mammalian ribosome biogenesis studies. As discussed below, aminoacridine-based compounds could also serve as prospective candidates for therapeutic inhibition of ribosome biogenesis. 

Incubation of cells with 9AA causes a rapid cessation of RNA Pol I transcription, leading to the disappearance of the 47S primary pre-rRNA transcripts within minutes after cell exposure to 9AA (Figure 1 and Figures S2 and S3). Treatment with 9AA did not induce detectable levels of shortened Pol I transcripts, which were observed to accumulate after treatments with ActD, a DNA intercalator commonly used as an inhibitor of rRNA synthesis [17,45]. Exposure of cells to the low doses of ActD was previously shown to impair Pol I elongation but not polymerase binding to DNA or initiation, resulting in multiple rounds of abortive elongation [17]. The binding of ActD is unusual in that it requires deoxyguanosine residues [46] and was also proposed to favor the altered DNA conformation present in the transcription complexes [47]. In contrast, when assayed in previous in vitro transcription studies of bacterial and phage polymerases, acridines were found to inhibit initiation by these enzymes [33]. Further study will be needed to elucidate the detailed mechanism of action of 9AA toward the Pol I transcription machinery in mammalian cells. It would also be interesting to compare 9AA mechanistically to other known inhibitors of Pol I-mediated transcription, such as CX-5461 and BMH-21 [20,21,25,26,27]. From a practical standpoint, as a readily available and inexpensive reagent, 9AA can circumvent the shortcomings of ActD in applications where a rapid and complete inhibition of synthesis of pre-rRNA is required. 

Our results also demonstrate that 9AA can inhibit maturation of pre-rRNA. Metabolic labeling of 9AA-treated cells revealed a diminished flux of the radioactive label from the already synthesized pre-rRNA transcripts to mature rRNA species (Figure 3). Northern hybridizations have shown that processing of the 32S pre-rRNA is particularly sensitive to 9AA, whereas higher 9AA doses inhibit multiple processing steps, leading to the accumulation of several large pre-rRNA species (Figure 4). Notably, inefficient 32S processing caused by 9AA treatment was observed at lower doses than those that completely inhibited Pol I transcription or disrupted the nucleolus. Given that 9AA readily binds to RNA (Figure 5), its interference with RNA–RNA and RNA–protein interactions could conceivably account for its ability to disrupt pre-rRNA processing. In support of this idea, other nucleic acid-intercalating agents were previously observed to interfere with pre-rRNA processing [48]. No prominent defects in pre-rRNA processing were detected with ActD, which primarily forms complexes with double-stranded DNA, but not with RNA or RNA–DNA duplexes [46,49]. 

The antagonistic effect of the archetypal aminoacridine 9AA on pre-rRNA metabolism we demonstrate here extends the list of 9AA activities in living cells and adds an important modality to consider with regard to the biological properties of other acridine-based molecules. Previous studies have largely focused on the intercalation into DNA and associated inhibition of DNA topoisomerases as the biologically relevant acridine properties [29,50,51,52,53]. Some observations, however, also indicate that the effects on DNA metabolism cannot fully explain acridines’ biological activities. For example, 9AA was found to activate the p53 protein and inhibit NF-κB function in the absence of a detectable DNA damage response or inhibitory effects on topoisomerase II [54]. 

To date, aminoacridine-based drugs have been used primarily as antibacterial and antiparasitic agents [51]. More recently, aminoacridine-based compounds have generated interest as prospective chemotherapeutic agents for cancer treatment [36,55,56,57]. Although one potential drawback of DNA intercalators is a risk of inducing DNA damage [58,59], several tested aminoacridines were reported to have low genotoxicity [34,54], and drugs like quinacrine have a long history of clinical applications [60]. Moreover, derivatization of the nucleic acid-targeting acridine core can greatly alter its binding specificities and biochemical properties [29,32,51], providing opportunities for further optimizations. Thus, exploration of acridine-based molecules might facilitate the development of ribosome biogenesis inhibitors with desirable pharmacological properties, expanding therapeutic options based on the interference with this pathway. 

## 4. Materials and Methods

### 4.1. Reagents

9AA hydrochloride monohydrate (cat #A38401) was purchased from Sigma-Aldrich, St. Louis, MO, USA. A 10 mM stock solution of 9AA was prepared in deionized water, sterile filtered, and stored protected from light at 4 °C. Actinomycin D (cat # 114666) was obtained from Calbiochem (EMD Chemicals, San Diego, CA, USA) and ethidium bromide solution (cat # E1510) was from Sigma.

### 4.2. Cell Culture and Metabolic Labeling

NIH 3T3 and HT1080 cells were grown in DMEM (Cellgro, Manassas, VA, USA) supplemented with 10% calf serum (HyClone, Logan, UT, USA) and penicillin (100 IU)/streptomycin (100 µg/mL) (Mediatech, Inc., Manassas, VA, USA). To obtain a 9AA dose-response curve, 3T3 cells were seeded in a 96-well plate at 1 × 10^3^ cells per well, treated the next day with 9AA for 2 h, washed, incubated in drug-free medium for a further 72 h, and quantified with an Alamar blue assay performed as described previously [13]. For metabolic labeling of pre-rRNA, cells were seeded in 6-well plates 2 days prior to the experiment. Cells were incubated in phosphate-free DMEM (Cellgro) supplemented with dialyzed fetal bovine serum (Gibco, Thermo Fisher Scientific, Waltham, MA, USA) for 15–30 min as indicated, then [^32^P]-orthophosphate (PerkinElmer, Boston, MA, USA) was added to a final concentration of 60 mCi/mL, and cells were incubated for 30 min. The radioactive medium was removed, and cells were washed once and then refed with complete standard medium containing 9AA or ActD where indicated.

### 4.3. RNA Analysis

Total cell RNA was isolated with RNAzol RT (Molecular Research Center, Inc., Cincinnati, OH, USA). A total of 2–2.5 µg total RNA per lane was resolved on 1% formaldehyde agarose gels and transferred onto Hybond-N nylon membranes (GE Healthcare, Milwaukee, WI, USA). Northern hybridizations were performed as previously described [61]. Sequences of probes used for hybridizations are listed in Table S1. Membrane images were obtained on a Typhoon 9410 phosphor imager and analyzed using ImageQuant 5.0 software (Molecular Dynamics, Sunnyvale, CA, USA). Prism 9.2 (GraphPad Software, San Diego, CA, USA) was used for quantitative analyses and curve fitting.

### 4.4. Immunofluorescence

Cells cultured on glass coverslips were washed with PBS (Mediatech), fixed with 4% paraformaldehyde for 10 min, permeabilized with 0.05% Triton X-100 for 10 min, and blocked with 1% normal goat serum (Sigma) in PBS. Cells were incubated with anti-NPM (Thermo Fisher Scientific) or antifibrillarin (EnCor Biotechnology, Gainesville, FL, USA) antibodies diluted 1:3000 and 1:100, respectively, for 16 h at 4 °C. Secondary Alexa Fluor 594 antimouse antibodies (Thermo Fisher Scientific) were used at 1:600 for 1 h at room temperature. Nuclear DNA was counterstained with 2 µg/mL Hoechst 33342 for 5 min, and samples were mounted in Prolong Gold (Thermo Fisher Scientific) and analyzed using a Nikon C2 confocal microscope (Nikon Instruments, Melville, NY, USA). Images were processed with NIS Elements AR (Nikon).

### 4.5. Fluorescent Intercalator Displacement

All measurements were performed in 5 × SSC buffer pretreated with DEPC in a flat-bottom, opaque, 96-well plate. First, 8 nM EtBr was premixed with 100 ng/mL solution of salmon sperm DNA (Sigma) or baker’s yeast RNA (Worthington Biochemical, Lakewood, NJ, USA). Then 9AA was added at the indicated concentrations, samples were incubated at room temperature in the dark for 30 min, and the emission spectra were collected with 525 nm excitation using a Synergy HT spectrofluorometer (BioTek, Winooski, VT, USA).

## Data Availability

Not applicable.

## Supplementary Materials

The following are available online at https://www.mdpi.com/article/10.3390/ijms23031260/s1.
