# Supplementary material for: 9-Aminoacridine Inhibits Ribosome Biogenesis by Targeting Both Transcription and Processing of Ribosomal RNA
Source: Int J Mol Sci. 2022 Jan 23;23(3):1260. doi: 10.3390/ijms23031260 (PMC8836032; doi:10.3390/ijms23031260)
Supplement: Supplementary file 1 [file ijms-23-01260-s001.zip › ijms-1527091-supplementary.pdf]

International Journal of *Molecular Sciences*

Article

9-Aminoacridine inhibits ribosome biogenesis by targeting both transcription and processing of ribosomal RNA

Supplementary Materials

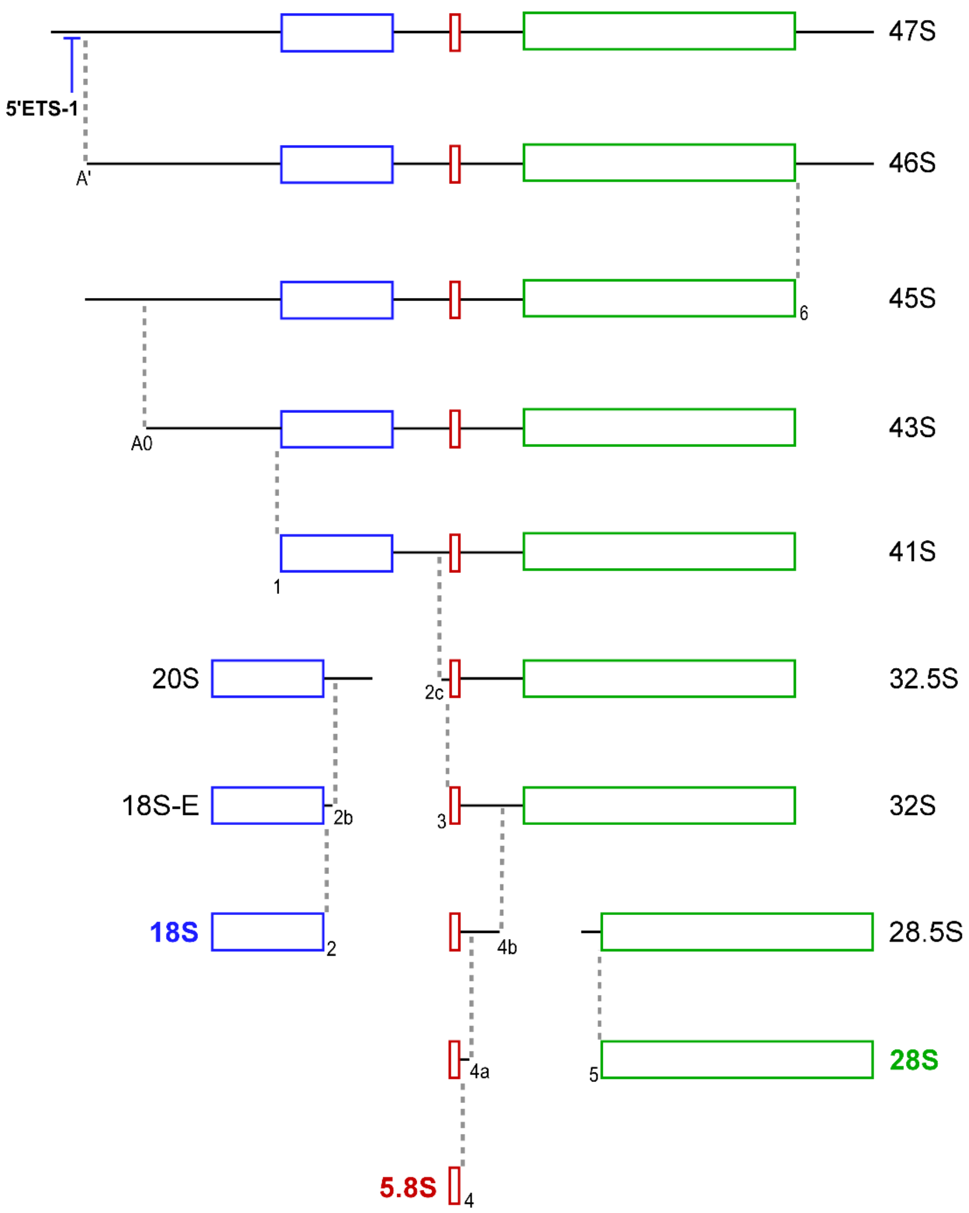

Figure S1. Mouse pre-rRNA processing intermediates. The primary 47S transcripts (top) can be detected with probe 5'ETS-1, which hybridizes to the 5' end removed during early processing at the A' site. Major precursors of 18S, 5.8S and 28S rRNA are shown, dotted lines mark processing sites.

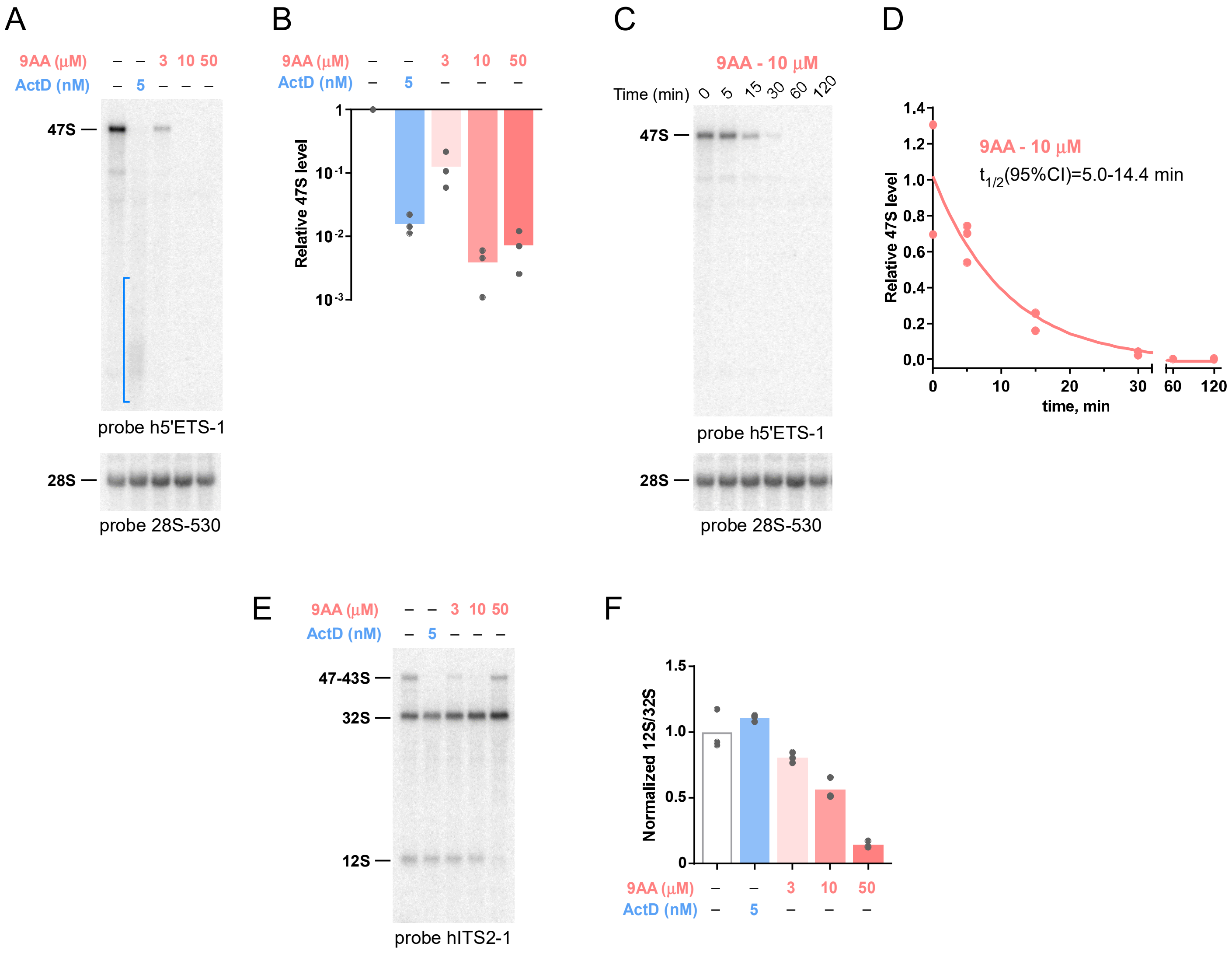

Figure S2. Effects of 9AA on pre-rRNA transcription and 32S pre-rRNA processing in human HT1080 cells. (**A**) Northern hybridization of RNA from cells treated with different concentrations of 9AA or ActD for 2 h. Products of Pol I transcription were visualized with probe h5'ETS-1 (top panel). The membrane was stripped and rehybridized with probe 28S-530 as a loading control (bottom panel). Bracket marks the smear of incomplete Pol I transcripts accumulating after ActD treatment. The experiment was repeated 3 times, a representative image is shown. (**B**) Relative abundance of the primary 47S pre-rRNA transcript. Hybridization signal of the full-length 47S was normalized by 28S rRNA in the same lane to correct for loading variation; bars show mean values obtained from 3 replicate cell cultures. (**C**) Representative time course of a 10 µM 9AA treatment. Northern hybridizations were performed as in (A). (**D**) Changes in the level of the primary 47S transcript over time after the addition of 9AA to cell medium. Data were obtained for 3 independent cell cultures and fit to a one-phase exponential decay equation. (**E**) Rehybridization of the membrane shown in (A) with probe hITS2-1. (**F**) Quantification of the hybridization signals of the 12S and 32S pre-rRNAs. The 12S/32S ratio in each lane was determined by phosphorimaging, bars show mean ratios for 3 replicate cell cultures.

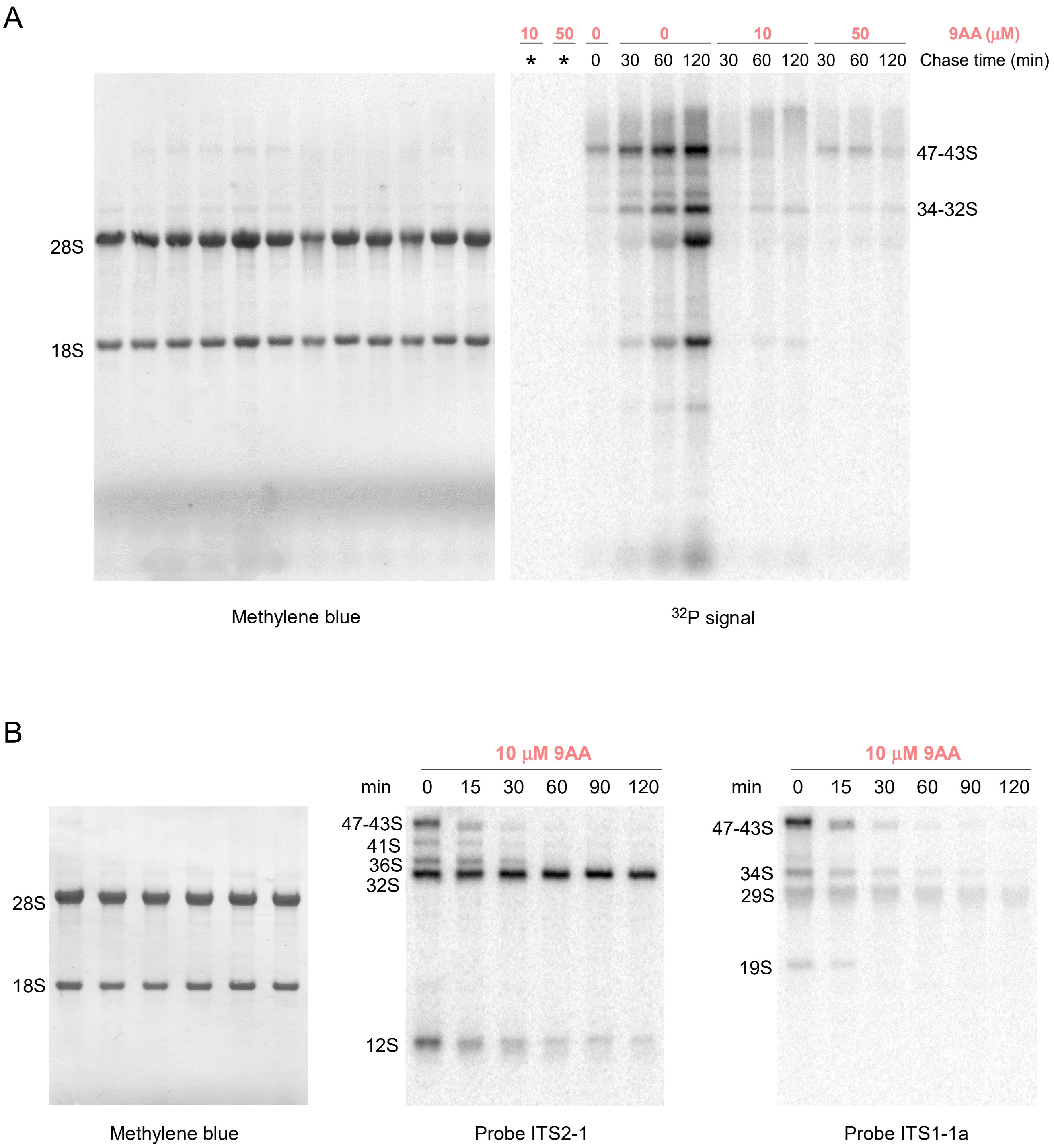

Figure S3. Additional pre-rRNA processing analysis in NIH 3T3 cells. (**A**) Cells were incubated in phosphate-free medium for 30 min, [^{32}P]-orthophosphate was added for 30 min, after which this labeling mix was replaced with complete, label-free medium. In the first two lanes (asterisk), 9AA was added 15 min *before* the 30-min incubation with [^{32}P]-orthophosphate and RNA was collected immediately after the labeling step. In all other lanes, 9AA was added together with the complete medium after the labeling step, RNA was isolated after different chase periods, resolved on an agarose gel, and transferred to a membrane. The [^{32}P] incorporated into RNA was detected by phosphorimaging (right). 18S and 28S rRNAs were stained on the same membrane with methylene blue to monitor loading (left). (**B**) Growing 3T3 cells were incubated with 9AA for 0-120 min. rRNA was analyzed by northern blotting as in Figure 4. Methylene blue staining of the membrane is shown on the left.

Table S1. List of oligonucleotide probes used in this study, **Uncropped gel images.**

**Name**	**Species**	**Sequence (5' to 3')**
5'ETS-1	mouse	AGAGAAAAGAGCGGAGGTTCGGGACTCCAA
5'ETS-3	mouse	AGCTCCCCACGGGAAAGCAATGAGTCTCTC
5'ETS-end	mouse	GGACAGAGAGCGCGAGAGAG
ITS1-1a	mouse	ACGCCGCCGCTCCTCCACAGTCTCCCGTT
ITS2-1	mouse	ACCCACCGCAGCGGGTGACGCGATTGATCG
3'ETS-1	mouse	AGAGCGACGGAAGGGGAAAGAGAAACGAAC
18S-500	mouse, human	GCAAATGCTTTCGCTCTGGT
28S-530	mouse, human	TTCTAAGTCGGCTGCTAGGC
h5'ETS-1	human	TAACGGAGGCAGAGACAGAG
hITS2-1	human	GGGTCTGCGCTTAGG
